# P2X Receptor Chimeras Highlight Roles of the Amino Terminus to Partial Agonist Efficacy, the Carboxyl Terminus to Recovery from Desensitization, and Independent Regulation of Channel Transitions*

**DOI:** 10.1074/jbc.M113.464651

**Published:** 2013-06-05

**Authors:** Rebecca C. Allsopp, Louise K. Farmer, Alistair G. Fryatt, Richard J. Evans

**Affiliations:** From the Department of Cell Physiology and Pharmacology, University of Leicester, Leicester LE1 9HN, United Kingdom

**Keywords:** ATP, Electrophysiology, Ion Channels, Mutagenesis, Pharmacology

## Abstract

P2X receptor subtypes can be distinguished by their sensitivity to ATP analogues and selective antagonists. We have used chimeras between human P2X1 and P2X2 receptors to address the contribution of the extracellular ligand binding loop, transmembrane segments (TM1 and TM2), and intracellular amino and carboxyl termini to the action of partial agonists (higher potency and efficacy of BzATP and Ap_5_A at P2X1 receptors) and antagonists. Sensitivity to the antagonists NF449, suramin, and PPADS was conferred by the nature of the extracellular loop (*e.g.* nanomolar for NF449 at P2X1 and P2X2-1EXT and micromolar at P2X2 and P2X1-2EXT). In contrast, the effectiveness of partial agonists was similar to P2X1 levels for both of the loop transfers, suggesting that interactions with the rest of the receptor played an important role. Swapping TM2 had reciprocal effects on partial agonist efficacy. However, TM1 swaps increased partial agonist efficacy at both chimeras, and this was similar for swaps of both TM1 and 2. Changing the amino terminus had no effect on agonist potency but increased partial agonist efficacy at P2X2-1N and decreased it at P2X1-2N chimeras, demonstrating that potency and efficacy can be independently regulated. Chimeras and point mutations also identified residues in the carboxyl terminus that regulated recovery from channel desensitization. These results show that interactions among the intracellular, transmembrane, and extracellular portions of the receptor regulate channel properties and suggest that transitions to channel opening, the behavior of the open channel, and recovery from the desensitized state can be controlled independently.

## Teaser

**Background:** P2X receptor subtypes show differences in pharmacology.

**Results:** Chimeric P2X receptors exhibit modified partial agonist action and recovery from desensitization.

**Conclusion:** Interactions among the extracellular, transmembrane, and intracellular regions regulate channel properties.

**Significance:** Agonist binding and transitions to channel opening (efficacy), behavior of the open channel, as well as recovery from desensitization can be controlled independently.

## Introduction

P2X receptors comprise a family of ATP-gated cation channels. The seven mammalian P2X receptor subunits (P2X1–7) have two transmembrane segments, intracellular amino and carboxyl termini, with a large extracellular ATP binding loop (1). P2X receptor subunits can assemble as homo- and heterotrimers, and 13 distinct recombinant receptor phenotypes have been described (2). At least one P2X receptor subtype is expressed by almost every cell type at some point during development. This widespread distribution of P2X receptors, and the variety of ways that extracellular levels of ATP can be increased *e.g.* in response to neuronal activity, mechanical stimulation/shear stress and tissue damage, result in P2X receptors making significant contributions to a range of physiological and pathophysiological conditions including muscle contraction, bone remodeling, and pain sensation (for review see Ref. 3).

The crystallization of the zebrafish P2X4 receptor (4, 5) has provided a quantum advance in understanding of agonist binding at the receptor and supported previous mutagenesis-based studies (6–8). A core ATP binding pocket consists of residues that are conserved throughout the P2X receptor family that co-ordinate the binding of the phosphate tail and adenine ring (4). These interactions have been proposed to contribute to the selectivity of the receptor for ATP over CTP, GTP, and UTP (4). The sensitivity and efficacy of ATP analogues vary considerably among receptor subtypes (1). For example α,β-methylene ATP (α,β-meATP)^2^ is equipotent with ATP at the human P2X1 receptor and has high efficacy, whereas at P2X2 receptors α,β-meATP is >100-fold less potent than ATP and has low efficacy (9). A chimera with the P2X2 receptor extracellular loop on a P2X1 receptor background or replacing either the first or second transmembrane segments of P2X2 with that from the P2X1 receptor increased agonist sensitivity to both ATP and α,β-meATP (10). However, whether the swaps of these regions had effects on other partial agonists, reciprocal effects in a mirror chimera, or the contribution of the intracellular regions of the receptor to regulating partial agonist efficacy were not determined. A naturally occurring rat and mouse P2X7 receptor splice variant (P2X7k) has an alternative amino terminus and part of the first transmembrane (TM1) segment. The P2X7k receptor has increased sensitivity to agonists (11) and increased efficacy of NAD-dependent ADP-ribosylation-mediated receptor activation compared with the P2X7a receptor (12, 13). This raises the possibility that intracellular regions of the receptor may also contribute to determining agonist action.

A key signature feature of P2X receptor subtypes is their time course. For example, P2X1 receptors show rapid transient responses that decay rapidly during continued ATP application (14) whereas at P2X2 receptors responses are relatively sustained (15). The study of a range of P2X receptor splice variants, point mutants, and chimeras (*e.g.* Refs. 16–19) has demonstrated the roles of both intracellular and transmembrane regions of the receptor in the regulation of time course of ATP responses. Recovery from the desensitized state takes several minutes, and agonist unbinding from the desensitized receptor contributes to recovery at P2X1 and P2X3 receptors (20, 21). However, less is known about which parts of the receptor are associated with recovery from the desensitized state following ATP removal.

We have recently generated a series of reciprocal chimeras between the human P2X1 and P2X2 receptors and demonstrated the important role of the pre-TM1 region in regulation of receptor time course (19). In this study we have used these chimeras to determine the contribution of the extracellular, transmembrane, and intracellular segments to partial agonist action and recovery from desensitization.

## EXPERIMENTAL PROCEDURES

### 

#### 

##### Generation of Chimeric P2X Receptors and Point Mutations

Chimeras were generated by mega-primer-mediated domain swapping as reported previously (19). In addition to chimeras, point mutants were made using the QuikChange mutagenesis kit (Stratagene). Production of the correct mutations and absence of coding errors was verified by DNA sequencing Automated ABI Sequencing Service, University of Leicester.

##### Expression in Xenopus laevis Oocytes and Electrophysiological Recordings

*X. laevis* oocytes were injected with transcribed sense strand cRNA (mMessage mMachine; Ambion) as described previously (19). Oocytes expressing WT P2X receptors were stored at 17 °C in ND96 buffer (96 mm NaCl, 2 mm KCl, 1.8 mm CaCl_2_, 1 mm MgCl_2_, 5 mm sodium pyruvate, 5 mm HEPES, pH 7.6) for 3–7 days. For electrophysiological recordings the 1.8 mm CaCl_2_ was replaced with 1.8 mm BaCl_2_. Two-electrode voltage clamp recordings were carried out in response to U-tube application (19) of agonists (all from Sigma).

##### Characterizing the Effects of Partial Agonists

The effectiveness of partial agonists was calculated as a percentage of the maximum ATP response (100 μm). Prior to application of partial agonists, reproducible ATP responses were established (time between applications ranged from 3 min for WT P2X2 receptors, increasing to a maximum of 20 min for those chimeras/point mutants showing slow recovery from desensitization). Concentration-response curves for partial agonists were normalized to the maximum ATP response at that receptor and fitted with the Hill equation (Equation 1),

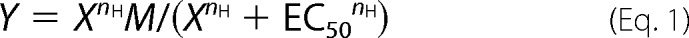
 where *Y* is response, *X* is agonist concentration, *n*_H_ is the Hill coefficient, *M* is the maximum response of the partial agonist expressed as a percentage of maximum response to ATP, and EC_50_ is the concentration of the agonist evoking 50% of the maximum response for that agonist. The pEC_50_ is given as −log_10_EC_50_ concentration. In the figures, concentration-response curves are fitted to the mean normalized data.

##### Characterizing the Effects of Antagonists

Antagonists were bath perfused for 5 min before being co-applied with an EC_90_ concentration of ATP. Inhibition curves were fitted with Equation 2,

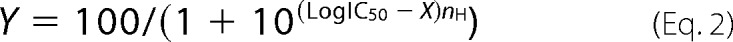
 where *Y* is % of control response, IC_50_ is the concentration of antagonist inhibiting the agonist control response by 50%, *X* is the log of the antagonist concentration, and *n*_H_ is the Hill coefficient.

##### Desensitization and Recovery from Desensitization of Chimeric Receptors

Desensitization of chimeric receptors and P2X receptor point mutants was assessed as described previously (19). Currents that decayed by 50% within a 20-s application of maximal ATP (100 μm) are described by “time to 50% decay ” (in seconds). For currents that did not decay by 50% during 20-s agonist application, percentage peak current remaining at the end of the 20-s application is given. For rapidly desensitizing responses “time to 50% recovery ” from desensitization was determined. To do this, reproducible currents to ATP (100 μm) were recorded (variable recovery times for reproducible maximum currents ranging from 3 to 20 min). ATP applications were then made at shorter time intervals in between reproducible recordings to determine time to 50% recovery from desensitization.

##### Statistical Analysis

Data are presented as mean ± S.E., and any differences between the means was determined by one-way analysis of variance followed by Bonferroni's test or Student's *t* test as appropriate. *n* refers to the number of oocytes tested.

## RESULTS

### 

#### 

##### Contribution of the Extracellular Loop to Agonist Action

**FIGURE 1.**
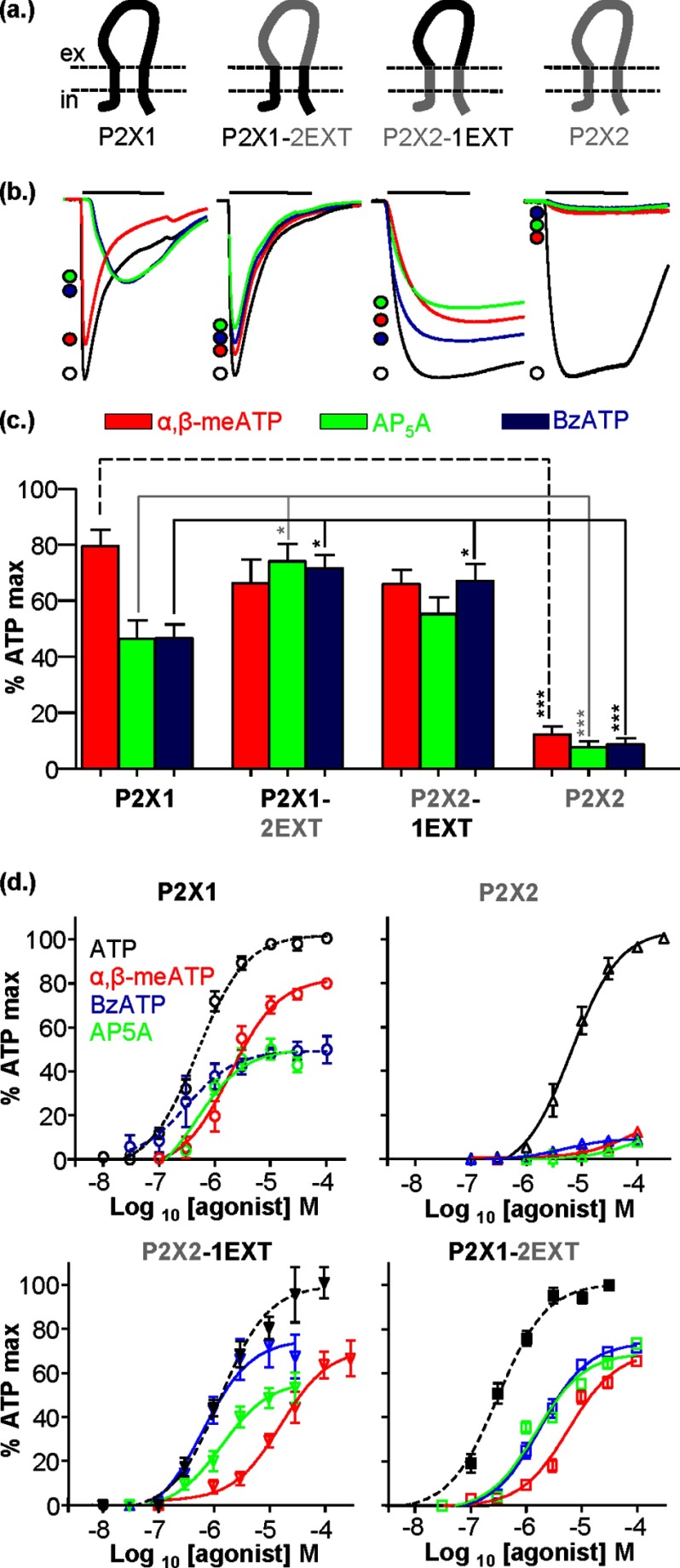
**Partial agonist sensitivity at P2X1 and P2X2 extracellular loop chimeras.** *a*, schematic representation of the P2X1 receptor (*black*), P2X2 receptor (*gray*), and receptor chimeras P2X1-2EXT and P2X2-1EXT generated by replacement of the entire extracellular loop. *b*, representative currents mediated by 3-s application (indicated by *bar*) of 100 μm ATP (*black*), α,β-meATP (*red*), AP_5_A (*green*), or BzATP (*blue*) to P2X1, P2X2, and chimeric receptors expressed in *Xenopus* oocytes. *c*, histogram summary showing the average peak current mediated by partial agonist application plotted as a percentage of maximum 100 μm ATP response. *d*, concentration-response curves (normalized to maximum ATP response) for ATP, α,β-meATP, BzATP, and AP_5_A at the P2X1, P2X1-2EXT, P2X2-1EXT, and P2X2 receptors. *Dotted line* represents concentration-response curves published previously (19, 41). *, *p* < 0.05; ***, *p* < 0.001 (*n* = 4–8). *Error bars*, S.E.

At hP2X1 receptors, ATP (100 μm, a maximal concentration) evoked inward currents that desensitized during the continued presence of agonist (Fig. 1). The analogues α,β-meATP, Ap_5_A, and BzATP were partial agonists with maximal efficacies at 100 μm of 80 ± 6, 46 ± 7, and 47 ± 5% of the maximum response to ATP, respectively (Fig. 1) similar to that reported previously (22). In contrast, at the hP2X2 receptor, ATP was ∼13-fold less potent, and the analogues were significantly less effective (12 ± 3, 8 ± 2, and 9 ± 2% maximum response to ATP for α,β-meATP, Ap_5_A, and BzATP, respectively, and EC_50_ values of >100 μm, Fig. 1). The structure of the ATP-bound zfP2X4 receptor showed the core residues that make the ATP binding site, and these are conserved throughout the P2X receptor family (4). Therefore, what determines the differences in ATP sensitivity and partial agonist action among receptor subtypes? Is it variations in the extracellular domain and/or changes in gating mediated by other parts of the receptor? To address this question we have characterized the action of ATP and the partial agonists at chimeras swapping the extracellular region of human P2X1 and P2X2 receptors.

At the chimeric receptor replacing the extracellular loop of the P2X1 receptor with that from the P2X2 receptor (P2X1-2EXT) ATP evoked currents of time course and sensitivity similar to those of the WT hP2X1 receptor (pEC_50_ 6.5 ± 0.1 and 6.3 ± 0.1 for P2X1-2EXT and WT hP2X1 receptor, respectively (19)). The efficacy of α,β-meATP at the P2X1-2EXT receptor (66 ± 8% of maximal response to ATP) was the same as the WT P2X1 receptors (80 ± 6%). However, the effectiveness of Ap_5_A and BzATP was increased to 74 ± 6 and 72 ± 5 of the maximal response to ATP (*p* < 0.05 for both). The pEC_50_ values for these partial agonists were 5.2 ± 0.1, 5.8 ± 0.1, and 5.7 ± 0.1 for α,β-meATP, Ap_5_A, and BzATP, respectively (compared with the <4 estimate at the P2X2 receptor) (Fig. 1). The finding that the properties of the P2X1-2EXT chimera are more similar to the P2X1 than the P2X2 receptor indicates that residues required for agonist recognition are present for both P2X1 and P2X2 receptors, as suggested previously for α,β-meATP (23). This suggests that interactions of the loop with the rest of the receptor make a significant contribution to the differences in agonist sensitivity and efficacy.

When the P2X2 receptor loop was replaced with that from the P2X1 receptor the ATP potency was intermediate between the P2X1 and P2X2 receptors (decreased ∼3-fold compared with P2X1 and increased ∼5-fold compared with the WT P2X2 receptor, *p* < 0.05 and *p* < 0.05, respectively). α,β-meATP sensitivity was also reduced at P2X2-1EXT compared with the P2X1 receptor (pEC_50_ values of 4.8 ± 0.2 and 5.7 ± 0.1, respectively, *p* < 0.01), but efficacy was unaffected. BzATP efficacy was increased by ∼10% for P2X2-1EXT compared with the P2X1 WT (*p* < 0.05), but this did not result in a change in sensitivity (pEC_50_ values of 6.5 ± 0.2 and 6.2 ± 0.2, respectively). The effectiveness of Ap_5_A was the same for P2X1 and P2X2-1EXT receptors as was the sensitivity to the agonist (pEC_50_ value 6.3 ± 0.2 and 5.9 ± 0.1, respectively). These findings with the P2X2-1EXT chimera suggest that agonist binding to the P2X1 receptor extracellular loop is more efficient at activating the transmembrane and intracellular parts of the P2X2 receptor than the P2X2 receptor loop. Taken together with the P2X1-2EXT chimera properties these results suggest that interactions of the extracellular loop with the rest of the receptor play an important role in regulating agonist sensitivity and efficacy.

##### Antagonist Sensitivity Is Determined by the Extracellular Loop

**FIGURE 2.**
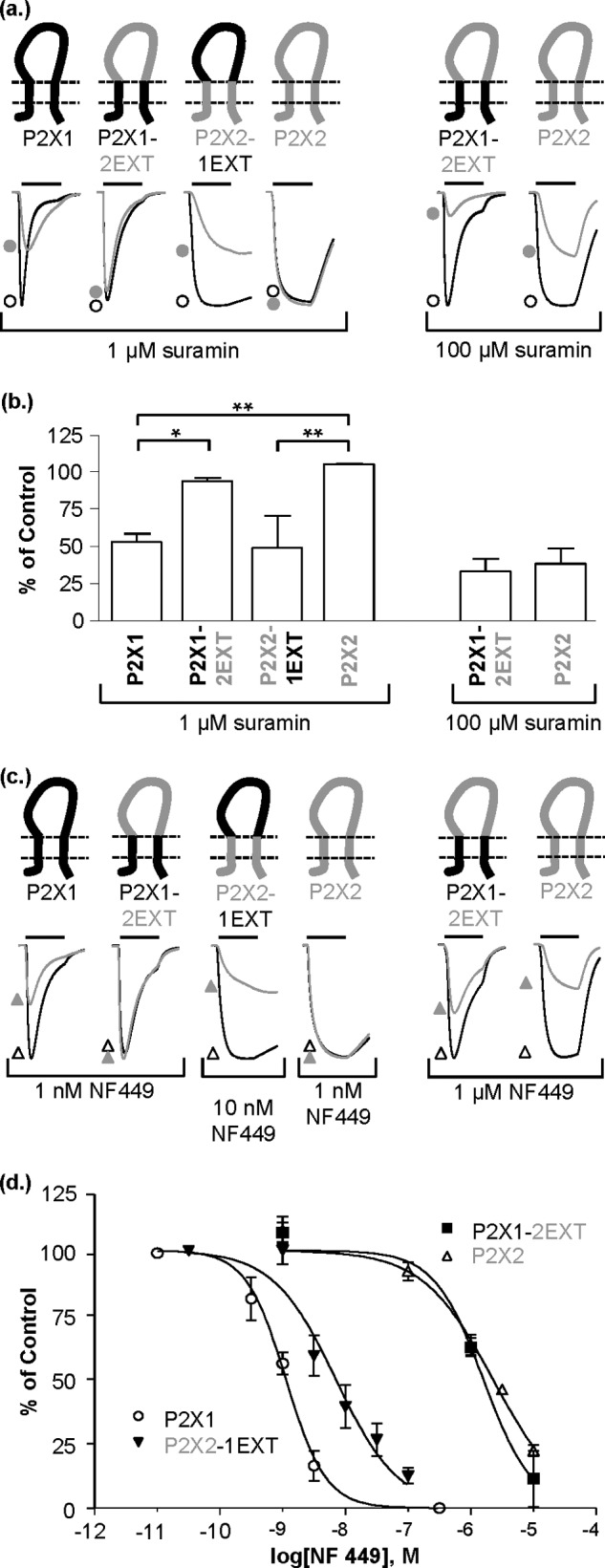
**Antagonist sensitivity tracks with the extracellular loop.** *a*, schematic showing P2X1 (*black*), P2X2 (*gray*), and chimeras P2X1-2EXT and P2X2-1EXT. Representative current traces mediated by a 3-s application (indicated by *bar*) of an EC_90_ concentration of ATP (*open circles*) and ATP plus suramin (*filled circles*) were recorded from *Xenopus* oocytes. *b*, histogram summary showing percentage of control current when suramin was presuperfused and co-applied with ATP. *Error bars*, S.E. *c*, representative current traces in response to an EC_90_ concentration of ATP (*open triangles*) and ATP plus NF449 (*filled triangles*). *d*, inhibition curves showing the effects of NF449 on WT and chimeric receptors. *, *p* < 0.05; **, *p* < 0.01 (*n* = 3–6).

The P2X1 and P2X2 receptors show differences in sensitivity to the antagonists suramin, NF449, and PPADS (measured against a standardized EC_90_ concentration of ATP; 1 μm at P2X1 and 100 μm at P2X2 receptors). Suramin was ∼100-fold more effective at hP2X1 than hP2X2 receptors (IC_50_ of ∼1 and 100 μm for P2X1 and P2X2, respectively, Fig. 2, *a*, and *b*). For the chimeras, the suramin sensitivity was determined by the nature of the extracellular loop with ∼50% inhibition of ATP response by 1 μm suramin at P2X2-1EXT receptors and ∼100 μm suramin at the P2X1-2EXT chimera (Fig. 2, *a* and *b*). The suramin derivative NF449 is a selective antagonist at P2X1 receptors (24), and we have shown previously an ∼1,500-fold difference in sensitivity between hP2X1 and hP2X2 receptors (25). Replacing the extracellular loop of the P2X1 receptor with that from P2X2 (P2X1-2EXT) produced a receptor that had low sensitivity to NF449 (pIC_50_ 5.8 ± 0.1). In the reciprocal chimera (P2X2-1EXT) NF449 had a pIC_50_ of 8.4 ± 0.2 similar to that of the WT P2X1 receptor (Fig. 2, *c* and *d*).

**FIGURE 3.**
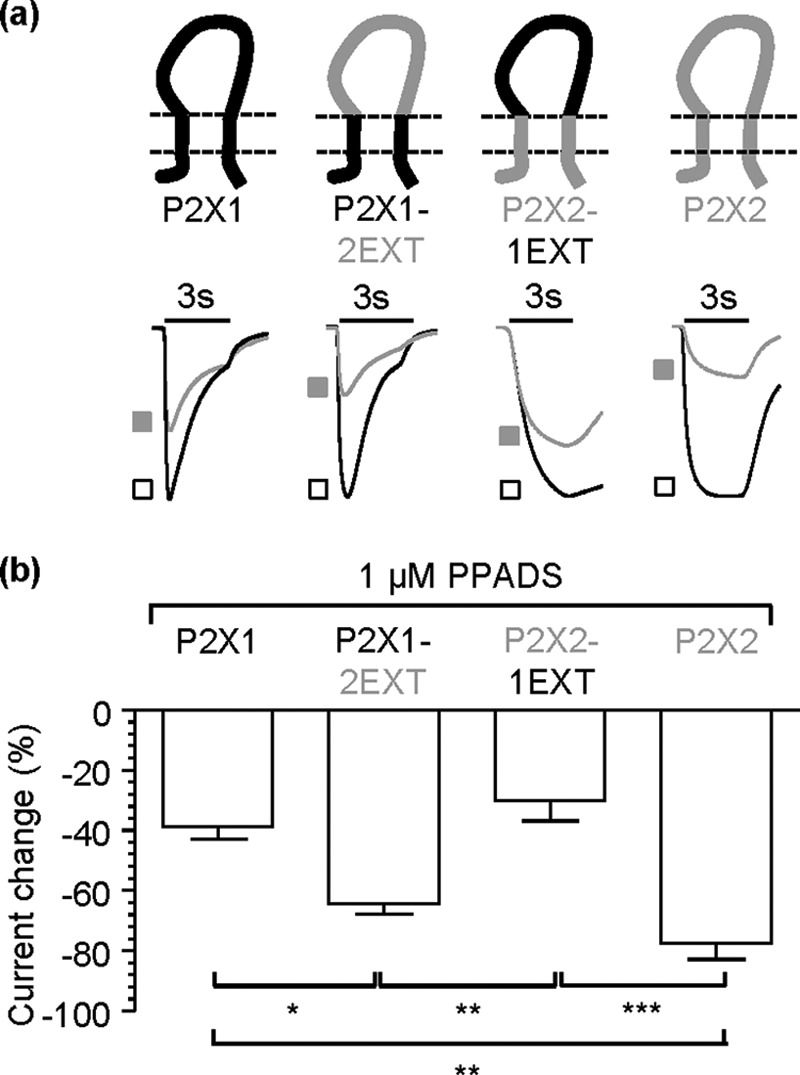
**PPADS sensitivity tracked with the extracellular loop.** *a*, schematic showing P2X1 (*black*) and P2X2 (*gray*) and chimeras P2X1-2EXT and P2X2-1EXT. Representative current traces mediated by a 3-s application (indicated by *bar*) of an EC_90_ concentration of ATP (*open squares*) and ATP plus PPADS (*filled squares*) were recorded from *Xenopus* oocytes. *b*, histogram showing percentage current change when PPADS was co-applied with ATP. *, *p* < 0.05; **, *p* < 0.01; ***, *p* < 0.001 (*n* = 3–5). *Error bars*, S.E.

There is a modest difference in PPADS sensitivity between hP2X1 and hP2X2 receptors. PPADS (1 μm) was ∼2-fold more effective in inhibiting responses at hP2X2 compared with hP2X1 receptors (77.6 ± 5.3 and 38.8 ± 4.1% inhibition of a standardized EC_90_ concentration of ATP for hP2X1 and hP2X2, respectively). This difference in inhibition was associated with the extracellular loop as when the loop of P2X1 receptor was replaced with that from P2X2 (P2X1-2EXT) the level of inhibition by PPADS (1 μm) was equivalent of that for the P2X2 receptor, and the converse was found for the reciprocal P2X2-1EXT chimera (Fig. 3). Taken together, these results suggest that differences in sensitivity to the antagonists suramin, NF449, and PPADS arise from variations in the extracellular ligand binding region. These data also suggest that these chimeras do not result in major conformational changes in the extracellular region of the receptor because if this were the case we would not expect there to be such reciprocal changes in antagonist sensitivity for the chimeras.

##### The Transmembrane Segments Can Regulate Partial Agonist Action

**FIGURE 4.**
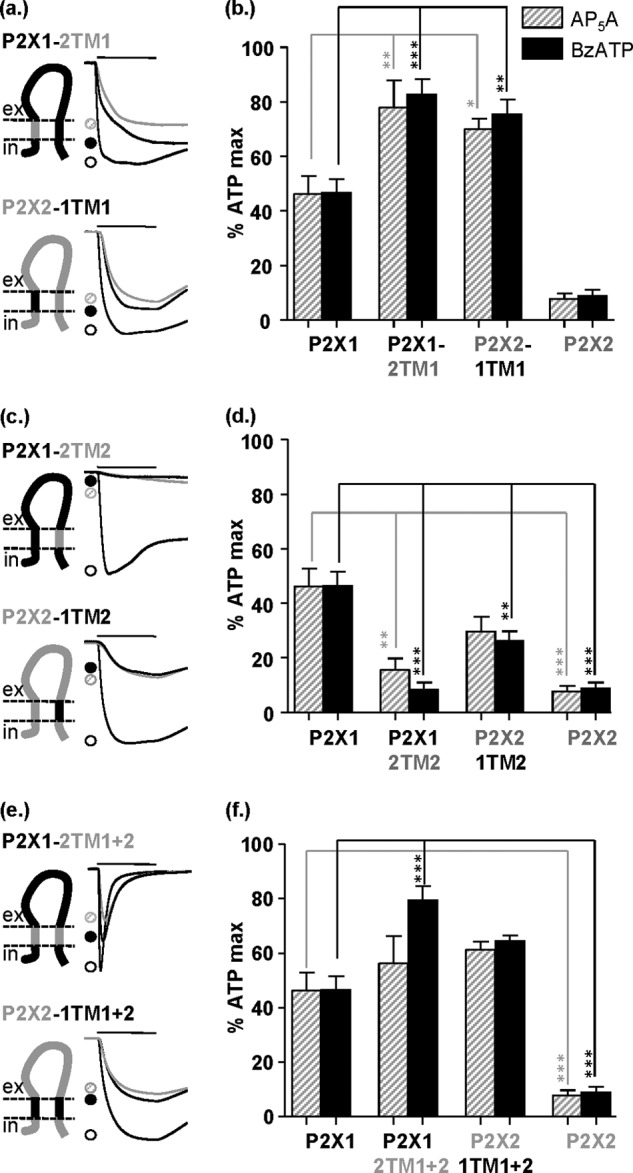
**Partial agonist sensitivity at P2X1 and P2X2 transmembrane domain chimeras.** *a*, *c*, and *e*, schematic representations of TM reciprocal chimeras and representative currents mediated by 3-s application (indicated by *bar*) of 100 μm ATP (*black open circles*), AP_5_A (*gray hatched circles*), or BzATP (*black filled circles*). *a*, TM1 chimeras. *c*, TM2 chimeras. *e*, TM1 + 2 chimeras. *b*, *d*, and *f*, histogram summaries showing the average peak currents mediated by partial agonist application plotted as a percentage of maximum 100 μm ATP response. *, *p* < 0.05; **, *p* < 0.01; ***, *p* < 0.001 (*n* = 4–8). *Error bars*, S.E.

The extracellular loop swap chimeras showed that the transmembrane segments and/or the intracellular regions could also have important roles in regulating partial agonist action. We have previously characterized the ATP sensitivity of the chimeras (19) but have not determined whether the chimeras have any effect on partial agonist action. To determine the contribution of the intracellular and transmembrane regions to partial agonist action we focused on Ap_5_A and BzATP because our previous work on P2X1 receptor mutants has shown that these are more sensitive to changes in agonist action than α,β-meATP (22). Previous studies showed that α,β-meATP action was increased at the chimeric rat P2X2-1TM1 receptor (10); however, the effects of other partial agonists or at the reciprocal chimera were not tested. In this study we see similar increases in effectiveness of the partial agonists Ap_5_A and BzATP (100 μm) at the hP2X2-TM1 chimeric receptor (to 70 ± 3.8 and 75 ± 5.6% maximal response to ATP respectively, Fig. 4), and this was associated with an increase in potency (pEC_50_ values to 4.6 ± 0.1 and 5.6 ± 0.1, respectively, compared with <4 for P2X2 WT, *n* = 3–8). The effectiveness of Ap_5_A and BzATP was also increased by ∼ 30% at the P2X1-2TM1 chimera (compared with P2X1 alone) (Fig. 4). However, this was not associated with a change in partial agonist sensitivity (pEC_50_ values Ap_5_A 6.3 ± 0.2, 6.2 ± 0.2, and BzATP 6.5 ± 0.2, 6.3 ± 0.2 for P2X1 WT and P2X1-2TM1 respectively, *n* = 3). This suggests that it is not the TM1 *per se* but its interactions with the rest of the receptor that are important for determining the effectiveness of partial agonists.

The second transmembrane segment (TM2) lines the ionic pore of the P2X receptor (4, 5). Mutations in this region have been shown to affect gating/spontaneous openings (26) so may play a role in the gating efficiency of partial agonists. Replacing TM2 of the P2X1 receptor with that from the P2X2 receptor (P2X1-2TM2) reduced partial agonist effectiveness to levels similar to the P2X2 receptor (Fig. 4), and so potency could not be estimated. We have shown previously that this chimera has no effect on ATP sensitivity (19). The chimera on the P2X2 receptor background (P2X2-1TM2) showed increased responses to the partial agonists to levels intermediate between P2X1 and P2X2 receptors. This was accompanied by an increase in sensitivity to ATP and the partial agonists (pEC_50_ values for ATP 5.7 ± 0.1, 5.2 ± 0.1, *p* < 0.001, Ap5A 4.2 ± 0.3, <4 estimate and BzATP 5.3 ± 0.1, <4 estimate for P2X2-1TM2 and P2X2 WT, respectively, *n* = 3–8). The reciprocal effects of TM2 swaps on partial agonist efficacy suggest a contribution of this region to partial agonist action.

When both transmembrane segments were replaced (P2X1-2TM1 + 2 and P2X2-1TM1 + 2) the efficacy of the partial agonists was equivalent to or slightly increased compared with WT P2X1 receptors (Fig. 4). For the P2X1-based chimera the increase in BzATP efficacy did not result in a change in potency (pEC_50_ values of 6.5 ± 0.2 and 6.4 ± 0.1 for P2X1WT and chimera, respectively, *n* = 3) (we have previously shown no effect on ATP potency (19)). The increase in efficacy of the partial agonists at the P2X2-1TM1 + 2 chimera to P2X1 receptor levels was associated with an increase in potency (6.5 ± 0.1 and 6.9 ± 0.1 for Ap5A and BzATP, respectively, *n* = 3) to that equivalent of the P2X1 WT receptor and an ∼6-fold increase in ATP potency above P2X1 WT (7.1 ± 0.1 for P2X2-1TM1 + 2 chimera and 6.3 ± 0.1 for WT P2X1 (19), *p* < 0.001, *n* = 3). These results suggest that there is a complex interaction both between the TMs and with the rest of the receptor in regulating partial agonist sensitivity and efficacy.

##### Regulation of Partial Agonist Action by Intracellular Regions

**FIGURE 5.**
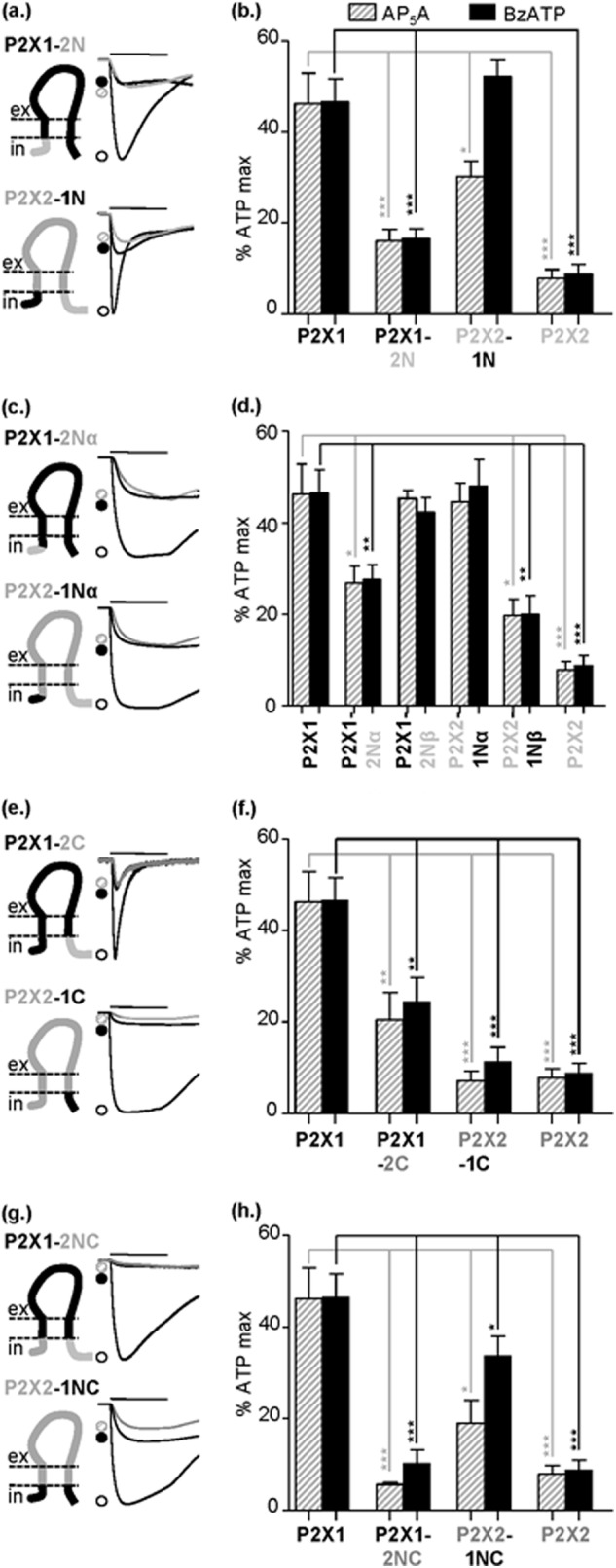
**Partial agonist sensitivity at P2X1 and P2X2 intracellular domain chimeras.** *a*, *c*, *e*, and *g*, schematic representations of amino- and carboxyl-terminal reciprocal chimeras and representative currents mediated by 3-s application (indicated by *bar*) of 100 μm ATP (*black open circles*), AP_5_A (*gray hatched circles*), or BzATP (*black filled circles*). *a*, amino-terminal chimeras. *c*, amino-terminal chimera subdivisions. *e*, carboxyl-terminal chimers. *g*, amino- and carboxyl-terminal chimeras. *b*, *d*, *f*, and *h*, histogram summaries showing the average peak current mediated by partial agonist application plotted as a percentage of maximum 100 μm ATP response. *, *p* < 0.05; **, *p* < 0.01; ***, *p* < 0.001 (*n* = 4–8). *Error bars*, S.E.

The intracellular regions of the P2X receptor are involved in regulation of the time course of ATP-evoked responses. We now show that efficacy of the partial agonists at the P2X1-2N chimera was reduced toward P2X2 receptor levels, and replacing the amino terminus of P2X2 with that from the P2X1 receptor (P2X2-1N) increased efficacy to P2X1 receptor levels (Fig. 5, *a* and *b*), suggesting that the amino terminus plays an important role in regulation of partial agonist efficacy. The decrease in efficacy for the partial agonists at P2X1-2N was not associated with a change in partial agonist sensitivity (pEC_50_ values of 6.5 ± 0.2 and 6.0 ± 0.3 for BzATP and 6.3 ± 0.2 and 6.2 ± 0.2 for Ap_5_A at P2X1 and P2X1-2N, respectively, *n* = 3). At the reciprocal chimera full concentration-response curves could not be generated because responses had not saturated at the highest concentration of partial agonist tested (100 μm). However, the responses at P2X2 and P2X2-1N had similar thresholds (10 μm for BzATP and 30 μm for Ap_5_A), indicating that there had been no major change in sensitivity. These results are consistent with our previous study showing that swapping the amino terminus between P2X1 and P2X2 receptors had no effect on ATP potency (19) and suggest that partial agonist potency and efficacy are differentially regulated.

Making chimeras that subdivided the amino terminus showed that residues 1–16 (Nα region) predominate in the regulation of efficacy with reciprocal effects of swapping this region between P2X1 and P2X2 receptors (Fig. 5, *c* and *d*). This region of the receptor is highly variable among P2X receptor subtypes. Swapping the second half of the amino terminus (residues 16–30, Nβ) had no effect on the P2X1 receptor background (P2X1-2Nβ) and gave a modest ∼2–fold increase in efficacy at the P2X2-1Nβ receptor. The chimeras swapping smaller regions of the amino terminus did not have as great an effect as changing the whole amino terminus. This suggests that although the first 16 amino acids predominate, residues in the second half of the amino terminus are also likely to fine-tune agonist efficacy.

Replacing the carboxyl terminus of the P2X2 receptor with the corresponding region of the P2X1 receptor (P2X2-1C) had no effect on partial agonist efficacy compared with the parent P2X2 receptor. In contrast, the P2X1-2C chimera had partial agonist efficacy intermediate between P2X1 and P2X2 receptors (Fig. 5, *e* and *f*). This reduction in efficacy was associated with a decrease in potency, which was estimated from the rightward shift in the threshold concentration for a response ∼100- and ∼300-fold for Ap_5_A and BzATP (full concentration-response curves had not saturated at the maximal concentration of agonist tested). The reduction in partial agonist potency is consistent with the reduction in ATP sensitivity we have reported for this chimera (19). The chimera with both intracellular regions of the P2X2 receptor (P2X1-2NC) showed partial agonist efficacy equivalent to that of the P2X2 receptor, and the reciprocal chimera (P2X2-1NC) had partial agonist efficacy intermediate between the P2X1 and P2X2 receptors (Fig. 5, *g* and *h*). This suggests that the intracellular regions of the receptor (predominantly the amino terminus) can play an important/dominant role in determining partial agonist potency.

##### Contribution of the Carboxyl Terminus to Recovery from Desensitization

**FIGURE 6.**
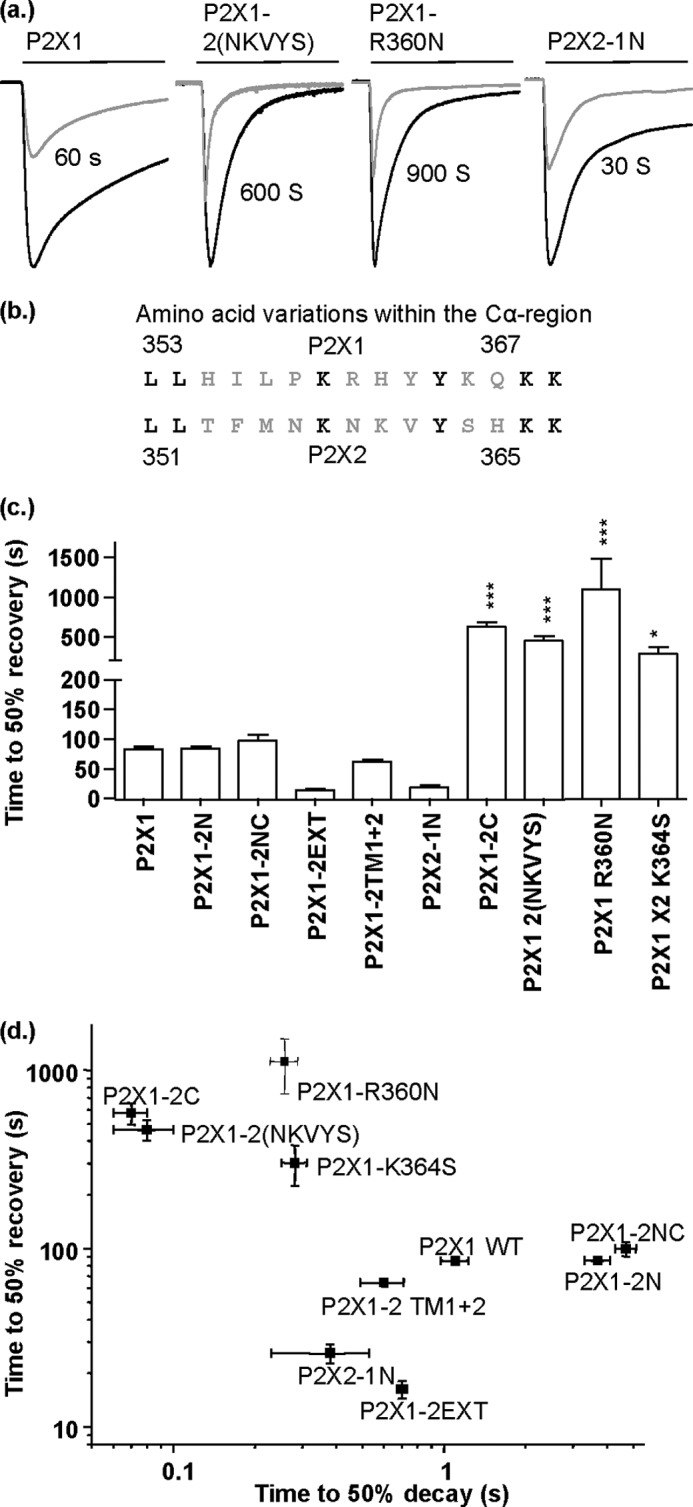
**Carboxyl terminus contribution to recovery from desensitization.** *a*, representative currents mediated by 3-s application (indicated by *bar*) of 100 μm ATP (*black traces*) and subsequent repeat application (time interval as indicated) resulting in approximately 50% recovery from desensitization compared with stable, reproducible, maximum ATP response. *b*, amino acid sequence line up showing the initial residues of the carboxyl-terminal section of P2X1 (*top*) and P2X2 (*bottom*). Mutations are based on the nonconserved amino acid residues within the Cβ region (shown in *gray*). *c*, histogram summary showing the time required in between successive, maximal, reproducible recordings (3-s 100 μm ATP applications) to achieve 50% recovery from desensitization. *d*, scatter plot showing the time to 50% recovery from desensitization after 3-s 100 μm ATP application *versus* time to 50% decay as measured over a 3-s or 20-s continuous 100 μm ATP application. *, *p* < 0.05; ***, *p* < 0.001 (*n* = 5–17). *Error bars*, S.E.

Of the chimeras tested four of them showed faster desensitization than the WT P2X1 receptor (P2X1-2EXT, P2X1-2C, P2X1-2TM1 + 2, and P2X2-1N). We were therefore interested to see whether the chimeras also had any effect on the time required for the response to recover from desensitization following agonist washout (Fig. 6). For WT P2X1 receptor responses to ATP (100 μm, a maximal concentration) the time to 50% recovery of the peak current to a subsequent application of ATP was ∼85s. The recovery time from desensitization was essentially the same for the chimeras P2X1-2EXT, P2X1-2TM1 + 2, and P2X2-1N (and for P2X1-2N and P2X1-2NC which showed a ∼3–4-fold slowing in desensitization) (Fig. 6*c*). In contrast, the most rapidly desensitizing chimera P2X1-2C (15-fold faster desensitization) required ∼10 min for the response to recover by 50% from desensitization. We have shown previously that swapping variant residues RHYYK to NKVYS (360–364) in the carboxyl terminus can account for the speeding in response of the P2X1-2C chimera (19). We now show that the P2X1-2NKVYS also shows prolonged recovery from desensitization, as do the point mutations P2X1-R360N and P2X1-K364S (464 ± 61, 1,118 ± 380, 302 ± 78; *p* < 0.001, *p* < 0.001, and *p* < 0.05, respectively). These results suggest that the carboxyl terminus of the P2X1 receptor plays a role not only in regulating the time course of the response but also the recovery from the desensitized state.

## DISCUSSION

P2X receptor subtypes can be distinguished based on their agonist and antagonist profiles. In this study we show that antagonist sensitivity was determined by the nature of the extracellular loop with nanomolar sensitivity to NF449 for the P2X1 receptor and the P2X2-1EXT chimera and micromolar sensitivity for the P2X2 receptor and P2X1-2EXT chimera. These studies demonstrate that variations in the amino acid sequence of the extracellular loop account for the differences in antagonist sensitivity between P2X receptors and support previous studies using point mutations and chimeras (25, 27–31).

There are marked differences in agonist action between P2X1 and P2X2 receptors (14, 15). We and others have shown previously that ATP potency was increased ∼20-fold when the P2X2 extracellular loop was placed on a P2X1 receptor background (P2X1-2EXT) and was equivalent to that of the P2X1 receptor (10, 19). We have now extended on these previous chimeric studies and show for the reciprocal chimera P2X2-1EXT ATP potency was intermediate between those of the P2X1 and P2X2 WT receptors. For the partial agonists there was an increase in sensitivity relative to ATP when the P2X2 loop was on a P2X1 receptor background (P2X1-2EXT) whereas the relative potencies of ATP and the partial agonists were similar for the P2X1 receptor and P2X2-1EXT chimera. Interestingly, the efficacy of the partial agonists was similar at both the loop swap chimeras. The exchange of sensitivity to the competitive antagonists suramin and NF449 with the extracellular loop chimeras indicates that there was no major conformational rearrangement around the agonist binding site that could account for these changes in agonist action.

The increased potency and efficacy of agonists at the P2X1-2EXT chimeras compared with the P2X2 receptor indicate that residues required for effective agonist binding are present at the P2X2 receptor extracellular loop, but that it couples less efficiently to the remainder of the P2X2 receptor compared with the P2X1 transmembrane and intracellular regions. In contrast, the P2X2-1EXT chimera had properties more similar to the P2X1 than P2X2 receptors, suggesting that the P2X1 loop couples more efficiently to the P2X2 receptor transmembrane and intracellular regions than the P2X2 loop. These findings also suggest that differences in the agonist binding site between the P2X1 and P2X2 receptor do not appear to play the defining role in determining either agonist sensitivity or efficacy. This reflects that residues forming the agonist binding pocket are highly conserved between the P2X receptor subunits (4). Our results support and extend previous chimeric and mutant studies (10, 19, 23, 32) and suggest that the gating properties imparted by interactions of the extracellular loop with the transmembrane and intracellular regions play a major role in determining both agonist sensitivity and the efficacy of the partial agonists.

The TM1 swap increased sensitivity to ATP and partial agonist action for P2X2-1TM1 and confirms and extends previous work on rat chimeras (10, 19). Interestingly, the partial agonists BzATP and Ap_5_A also showed increased effectiveness at the reverse chimera, P2X1-2TM1; however, this did not result from a change in partial agonist sensitivity. We have shown previously that the P2X1-2TM1 had an ∼10-fold increase in ATP sensitivity (19), suggesting different coupling of the ATP binding event from that for the partials (see below). In contrast, TM2 swaps showed reciprocal effects on partial agonist action (decrease at P2X1-2TM2 and increase at P2X2-1TM1), suggesting that TM2 contributes to partial agonist effectiveness differences between P2X1 and P2X2 receptors. However, when both TMs were swapped the effects on partial agonist action were equivalent to those of the TM1 swap alone (increased action). This indicates that variations in agonist action between P2X receptor subtypes are unlikely to be controlled by a signature amino acid sequence in the TMs and that it is the complex interaction between the TMs and the rest of the receptor that regulates partial agonist action.

**FIGURE 7.**
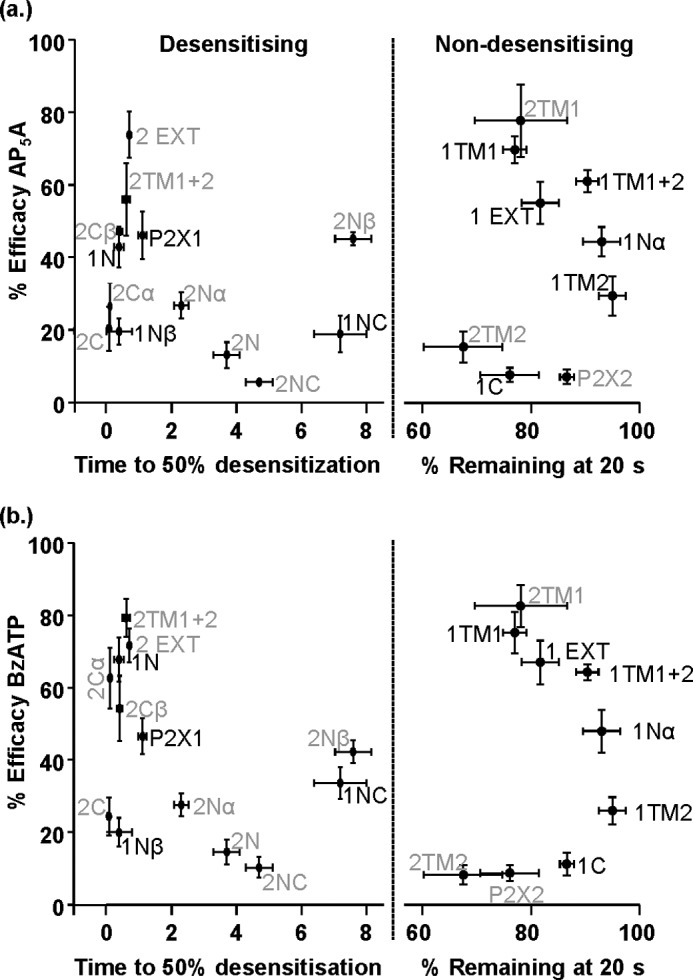
**Efficacy of partial agonists (compared with ATP) for WT and chimeric receptors shows no correlation with the time course of ATP-mediated currents.** Efficacy of partial agonists AP_5_A (*a*) and BzATP (*b*) as a percentage of maximum ATP evoked response (100 μm ATP) *versus* the receptor time course to desensitization is shown. Time course is expressed as the time to 50% desensitization over 3-s ATP application, or percentage current remaining at the end of prolonged 20-s ATP application, for fast/slow desensitizing receptors, respectively (*n* = 5–17).

The reciprocal effects on partial agonist efficacy of swapping the amino terminus between P2X1 and P2X2 receptors (decreased to P2X2 levels for P2X1-2N and increased to P2X1 levels for P2X2-1N) demonstrate the important role of the interaction of this region with the rest of the receptor in determining receptor properties. This is consistent with previous studies at P2X2 receptors showing the role of the amino terminus in regulation of the time course of P2X receptor properties (18) and the demonstration of conformational movement of the amino terminus in response to agonist (33). The changes in efficacy to BzATP and Ap_5_A in the present study were not accompanied by any change in their sensitivity, and this is consistent with our previous work reporting no effect on ATP sensitivity of these chimeras (19). Our results show that agonist potency and efficacy can be controlled independently at P2X receptors. Several of the chimeras used in the present study had an effect on the time course of agonist evoked currents. However, analysis of the efficacy of the BzATP or Ap^5^A for the range of chimeras tested showed no correlation with the time course of ATP evoked currents (Fig. 7), demonstrating that efficacy and time course can also be regulated independently. This is perhaps not unexpected because efficacy can be considered as the ability to bind to and activate the channel whereas the time course reflects that behavior of the channel once it has opened.

**FIGURE 8.**
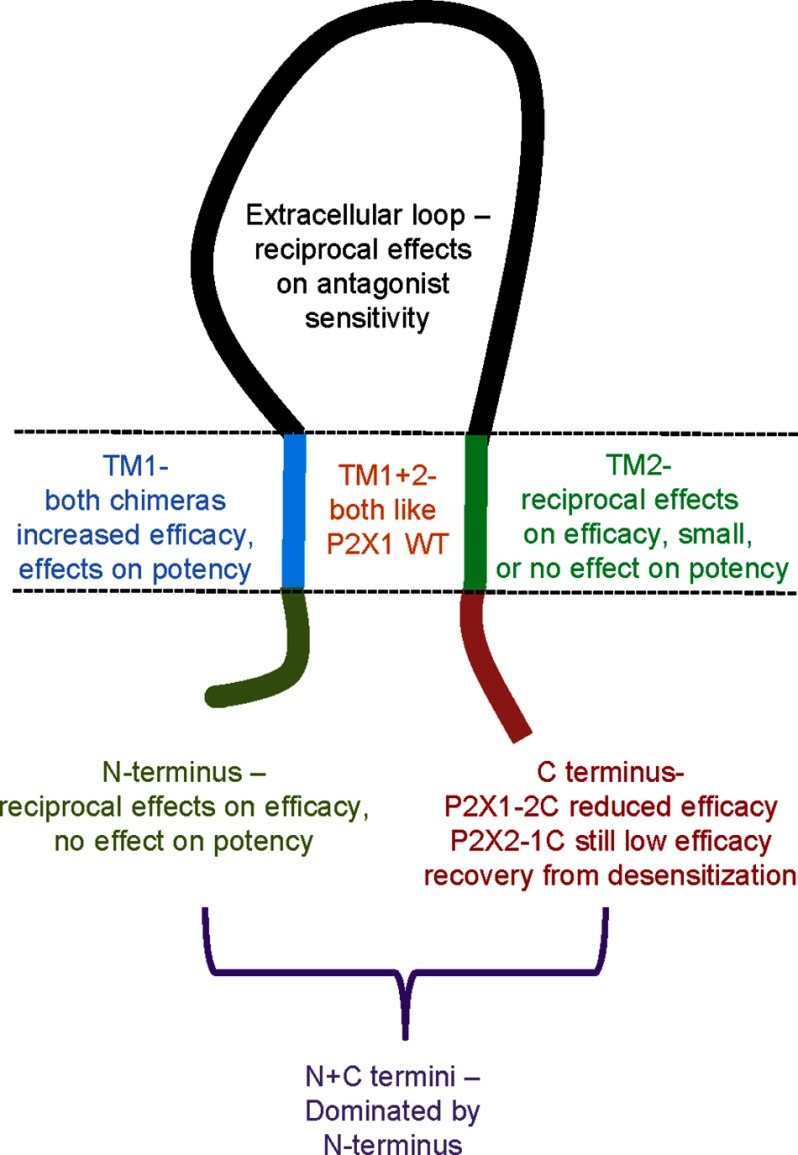
**Summary of effects of chimeras on partial agonist potency and efficacy.** Replacement of different regions of the P2X1 receptor with those from the P2X2 receptor, and vice versa modified partial agonist action. Residues in the extracellular loop determine antagonist sensitivity. The TM regions are involved in control of both agonist sensitivity and efficacy. The amino terminus can regulate agonist efficacy independently of sensitivity, and the carboxyl terminus is involved in recovery of the receptor from the desensitized state.

The present study shows that interactions between different parts of the receptor can modify the ability of the partial agonist bound receptor to efficiently transition to the open state (efficacy) (for summary, see Fig. 8). Del Castillo and Katz proposed a two-step model of agonist action where the agonist (A) binds to the receptor (R) and from this agonist-bound state (AR) the channel is able to open (to the AR* state) (34). A partial agonist would have reduced efficacy if it was inefficient at eliciting the change in conformation between the agonist bound shut (AR) and open state (AR*). A chimera that decreased the energy required for/favored channel opening (the AR to AR* step) would lead to an increase in ATP sensitivity and increase both sensitivity and efficacy of the partial agonists. This appears to be the case for the P2X1-2EXT chimera (this study and Ref. 10). However, in the present study for several chimeras there was a change in partial agonist effectiveness without a concomitant change in potency (*e.g.* P2X1-2N and P2X2-1N). This suggests a more complex mechanism of ligand action at P2X receptors with a potential series of transitions involved in ligand binding and channel opening that can be differentially regulated for ATP and partial agonists. At glutamate channels structural studies on the extracellular ligand binding core show that it adopts a range of ligand-dependent conformations, and at the intact receptor partial agonists give rise to discrete single-channel properties (35). At the nicotinic receptor superfamily it has been suggested that after agonist binding there are a number of intermediate shut states before channel opening and that the response to partial agonists is limited by conformational changes in one of the shut states (36). ATP binding promotes closure of the agonist binding site that is located at an intersubunit cleft (4, 37). Whether partial agonists bind in the same way as ATP at P2X receptors and/or show different transitions through agonist-bound shut states remains to be determined. What is clear from the present study is that complex interactions among the intracellular, transmembrane, and extracellular portions of the receptor are involved in agonist action.

Receptor desensitization and recovery on removal of agonist have a major impact on receptor signaling. Previous studies have suggested that agonist unbinding from the desensitized receptor contributes to recovery from desensitization at P2X1 and P2X3 receptors (20, 31, 38). In this study we have shown that in addition the carboxyl terminus plays a key role in recovery from desensitization. For the P2X1 receptor-based chimera introduction of the carboxyl terminus of P2X2 (P2X1-2C) decreased ATP potency ∼4-fold (19) but increased time to 50% recovery ∼8-fold. These results support that recovery from desensitization is not regulated solely by agonist sensitivity/unbinding and that an independent conformational change may also be involved. Smaller changes in the carboxyl-terminal region identified point mutations R360N and K364S as the differences that could account for the slowing in recovery of the P2X1-2C chimera. Residues 360 and 364 are close to the TM2 pore-forming segment of the receptor, raising the possibility that at P2X1 receptors they interact with the pore forming TM2 segment to stabilize the desensitized receptor state. However, whether this corresponds to an effect on the time the receptor spends in an agonist-bound desensitized state prior to agonist unbinding and/or an agonist-free desensitized state remains to be determined.

In summary, we have shown that concerted interactions of the intracellular amino and carboxyl termini with other parts of the receptor regulate channel properties and that agonist binding and transitions to channel opening (efficacy), the behavior of the open channel, as well as recovery of P2X receptors from desensitized state can be independently controlled. As the intracellular regions of the zebrafish P2X4 receptor were removed to aid crystallization (4, 5) there is no structural information regarding these regions or their proximity to the rest of the receptor. FRET studies and antibody labeling showed that for the human P2X4 receptor the ends of the carboxyl termini are ∼60 Å apart (39), and concatenated P2X2 receptors are functional (40), suggesting that the amino and carboxyl termini from adjacent subunits may be close in the native receptor. However, further work is needed to determine the structure and extent of interactions of the intracellular portions of the receptor and how this may regulate channel properties.

## Footnotes

2 The abbreviations used are:

α,β-meATP: α,β-methylene ATP
Ap_5_A: P^1^,P^5^-diadenosine 5′-pentaphosphate
BzATP: 2′,3′-*O*-(4-benzoylbenzoyl)-ATP
EXT: extracellular loop
PPADS: pyridoxal-phosphate-6-azophenyl-2′,4′-disulfonate
TM1: first transmembrane
TM2: second transmembrane.
